# Characterizing the Neuroprotective Effects of S/B Remedy (*Scutellaria baicalensis* Georgi and *Bupleurum scorzonerifolfium* Willd) in Spinal Cord Injury

**DOI:** 10.3390/molecules24101885

**Published:** 2019-05-16

**Authors:** Tsung-Hsi Tu, Dann-Ying Liou, Di-You Lin, Hsin-Chun Yang, Ching-Jung Chen, Ming-Chao Huang, Wen-Cheng Huang, May-Jywan Tsai, Henrich Cheng

**Affiliations:** 1 Taiwan International Graduate Program in Molecular Medicine, National Yang-Ming University and Academia Sinica, Taipei 11217, Taiwan; thtu0001@gmail.com; 2 Department of Neurosurgery, Neurological Institute, Taipei Veterans General Hospital, Taipei 11217, Taiwan; dragonspruce@gmail.com (D.-Y.L.); commondio@gmail.com (D.-Y.L.); Hcy0402@gmail.com (H.-C.Y.); jirong.chen88@gmail.com (C.-J.C.); mchuang@vghtpe.gov.tw (M.-C.H.); wchuang@vghtpe.gov.tw (W.-C.H.); 3 School of Medicine, National Yang-Ming University, Taipei 11217, Taiwan; 4 Department and Institute of Pharmacology, National Yang-Ming University, Taipei 11217, Taiwan

**Keywords:** herbal remedy, Sho-Saiko-To, neuroprotection, anti-inflammation, spinal cord injury, neuronal/glial cultures

## Abstract

The main causes of dysfunction after a spinal cord injury (SCI) include primary and secondary injuries that occur during the first minutes, hours, to days after injury. This treatable secondary cascade provides a window of opportunity for delivering therapeutic interventions. An S/B remedy (*Scutellaria baicalensis* Georgi and *Bupleurum scorzonerifolfium* Willd) has anti-inflammatory, cytoprotective, and anticarcinogenic effects in liver or neurodegenerative diseases. The present work examined the effect of S/B on injured spinal cord neurons in cultures and in vivo. S/B effectively reduced peroxide toxicity and lipopolysaccharide stimulation in both spinal cord neuron/glial and microglial cultures with the involvement of PKC and HSP70. The effect of S/B was further conducted in contusive SCI rats. Intraperitoneal injections of S/B to SCI rats preserved spinal cord tissues and effectively attenuated microglial activation. Consistently, S/B treatment significantly improved hindlimb functions of SCI rats. In the acute stage of injury, S/B treatment markedly reduced the levels of ED1 expression and lactate and had a tendency to decrease lipid peroxidation. Taken together, we demonstrated long-term hindlimb restoration alongside histological improvements with systemic S/B remedy treatment in a clinically relevant model of contusive SCI. Our findings highlight the potential of an S/B remedy for acute therapeutic intervention after SCI.

## 1. Introduction

Traumatic spinal cord injury (SCI) is a multifactorial and devastating process which leads to loss of neurological function below the level of injury. The trauma involves a contusion, followed by various mechanisms of secondary injury cascades which include blood–brain barrier dysfunction, local inflammation, ischemia, neuronal death, demyelination and disrupted nerve pathways [1]. The outcome of SCI depends on the extent of secondary damage which occurs during the first minutes, hours, and days after injury [2,3]. Within two days after injury, blood-spinal cord barrier disruption and hemorrhage causes a rapid influx of inflammatory cells and cytokines [4]. Necrotic cell death releases ATP, potassium ions, and DNA, and activates microglia. Phagocytes generate free radicals and produce additional cell injury through protein/lipid oxidation and DNA damage [5,6]. This treatable secondary cascade provides a window of opportunity for the delivery of therapeutic interventions. Current treatment options for SCI are limited. The FDA-approved drug methylprednisolone (MP) as a therapeutic option is still a matter of debate. MP is usually administered in the acute phase at a high dose for 48 h [7]. However, its efficacy is controversial with several side effects, including increased risks of urinary tract and gastrointestinal hemorrhage, and a trend to increase overall adverse events [8].

Neuroprotective agents aiming to reduce further injury are potential key therapies in SCI. Herbal remedies are a promising prescription for various kinds of disease, including central nervous system (CNS) injury. Xiao-Tsai-Hu-Tang in mandarin, equivalent to Japanese Sho-Saiko-To (TJ-9), is one of the most frequently prescribed Kampo medicines and has been widely used for the treatment of various inflammatory disorders, particular chronic hepatitis [9,10,11]. Sho-saiko-to is the powder extracted by hot water from the mixture of seven medical herbs containing several bioactive ingredients such as saikosaponins, baicalin, baicalein, and glycyrrhizic acid [12]. According to the original proportion of herbal combination, a simplified remedy consists of two major components of Sho-saiko-to, *Scutellaria baicalensis Georgi* (Sb) and *Bupleurum scorzonerifolfium Willd* (Bs) (abbreviated as Sb/Bs with a ratio of 7 to 3, Sb to Bs in an S/B remedy), has been shown to have similar pharmacological effects on liver disease [13]. The S/B remedy is comprised of significant amounts of active flavonoids [9,14]. Compositional analysis of the S/B remedy by high performance liquid chromatography (HPLC) showed that the major components in S/B were baicalin and saikosaponin a-c, which were similar to Sho-Saiko-To. Flavonoids may exert a variety of biological actions such as antioxidant and anti-inflammatory activities, supporting the possible application of simplified form of Xiao-Tsai-Hu-Tang in acute or chronic nerve injury.

Traumatic injury to the spinal cord provokes a striking inflammatory response that results in further tissue damage [15,16]. Attenuation of the early inflammatory response to spinal cord injuries may therefore limit the extent of tissue injury, and accordingly, the consequent disability. Sb, one of component of S/B has been used in oriental medicine to treat inflammatory diseases [17,18] and was applied to spinal cord injury with positive outcomes [19]. The powder extract from the roots of Sb and Bs was also demonstrated to be effective in attenuating iron-induced damage in the nigrostriatal system [14]. These support the use of modified formula of Xiao-Tsai-Hu-Tang in treating CNS disease or nerve injury. However, no reports have ever examined their efficacy in injured spinal cord neurons. Accordingly, we hypothesized that the S/B remedy may be a promising remedy for victims after traumatic SCI. This project examined whether the S/B remedy could protect the complicated and devastating contusive spinal cord injury in rats and to understand the underlying mechanisms of its beneficial effects. We demonstrated the therapeutic effects of the S/B remedy in injured spinal cord in culture and in vivo. The S/B remedy effectively reduced lipopolysaccharide (LPS) stimulation and protected peroxide toxicity in spinal cord neuron-glial cultures and microglial cultures. In vivo study further showed that the S/B remedy reduced microglial activation and tissue damage and enhanced neurobehavioral recovery after traumatic SCI. Thus, the S/B remedy appears to be a promising therapeutic strategy for acute SCI.

## 2. Results

### 2.1. S/B remedy Possessed Anti-Inflammatory and Anti-Oxidative Activities in Spinal Cord Neuronal/Glial Cultures

**Figure 1:**
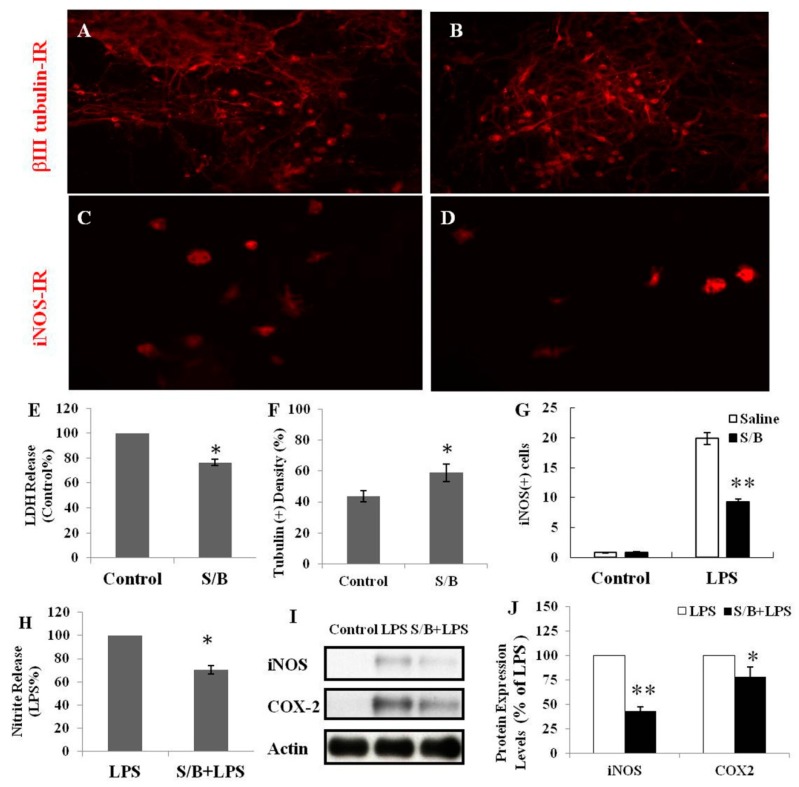
The effects of S/B remedy on cell survival or lipopolysaccharide (LPS) stimulation in spinal cord neuron/glial cultures, wherein (**A**) shows Control culture, tubulin-IR, (**B**) shows S/B-treated cultures, tubulin-IR, (**C**) shows LPS-treated culture, iNOS-IR, (**D**) shows S/B+LPS-treated cultures, iNOS-IR (**E**) shows LDH release in Control or S/B-treated cultures, (**F**) shows tubulin(+) density in Control or S/B treated cultures (F is the quantification of A and B), (**G**) shows iNOS-positive cells in each group of cells (G is the quantification of C and D), (H) shows the amount of nitrite release in each group of cells, (**I**) shows Protein expression of LPS- or S/B+LPS-treated cultures, and (**J**) shows quantitative results of iNOS or COX2 expression levels in I. The symbols “*” and “**” indicate statistical significance by one-way ANOVA and Bonferroni *t*-test at *p* < 0.05 (S/B vs. Control or S/B+LPS- treated vs. LPS-treated cells) and *p* < 0.01 (S/B + LPS-treated vs. LPS- treated cells), respectively.

**Figure 2:**
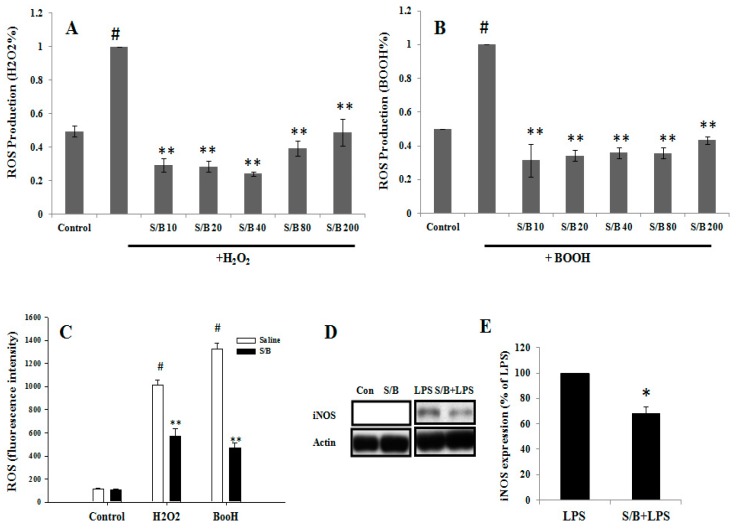
The effects of S/B remedy on H_2_O_2_- and tert-BOOH-induced free radical ROS formation and LPS stimulation in primary CNS cell cultures, wherein (**A**) shows H_2_O_2_ (1 mM)-induced ROS in spinal cord neuron-glia cultures and with S/B treatment for 2 h, (**B**) and shows tert-BOOH (0.75 mM)-induced ROS in spinal cord neuron-glial cultures and with S/B remedy treatment for 2 h. The symbols “S/B10”, “S/B20”, “S/B 40”, “S/B 80”, and “S/B 200” refer to the Sb/Bs at 10, 20, 40, 80, and 200 µg/mL, respectively. (**C**) shows H_2_O_2_ (3 mM)- or tert-BOOH (0.75 mM)-induced ROS in microglia cultures and with S/B remedy (80 µg/mL) treatment. (**D**) shows iNOS expression in Control, S/B-, LPS- or S/B+LPS-treated microglia. (**E**) shows quantitative results of iNOS expression in LPS- or S/B+LPS-treated microglia from three independent experiments. The symbols “#”, “*” and “**” indicate statistical significance by one-way ANOVA and Bonferroni t-test at *p* < 0.01 (H_2_O_2_ or tert-BOOH treated cells vs. control), *p* < 0.01 (peroxide plus S/B vs. peroxide alone) and *p* < 0.05 (LPS plus Sb/Bs vs LPS alone), respectively.

**Figure 3:**
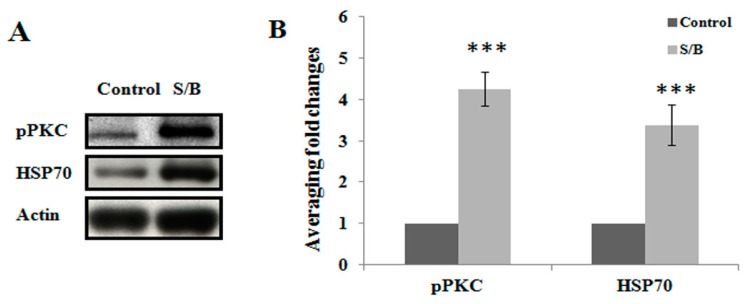
The effects of S/B remedy on levels of pPKC and HSP70 in neuronal/glial cultures, wherein (**A**) shows the protein expression of control or S/B-treated cultures, and (**B**) shows the quantitative results of pPKC or HSP70 expression levels in A. Data are expressed as means ± SEM from 5 independent experiments. The symbols “***” indicate statistical significance by one-way ANOVA and Bonferroni t-test at *p* < 0.001, S/B vs. Control.

We first examined the beneficial effect of the S/B remedy in spinal cord neuronal/glial cultures. Figure 1A,B,E,F shows that S/B remedy (10 µg/mL) treatment for 2 days could enhance cell survival in cultures, as evidenced by a reduction of LDH release and increased tubulin-immunoreactive (IR) density in S/B-treated cultures. Mimicking the inflammatory response in spinal cord after injury, we applied a strong immune challenger lipopolysaccharide (LPS, 1.2 µg/mL) in spinal cord cultures to induce inflammatory responses in the presence or absence of the S/B remedy (10 µg/mL). Two days later, the cells were harvested for immunohistochemical and protein expression assays, and the media were collected for determining the levels of nitrite and LDH release. Figure 1C,D,G,H show that LPS-induced increases of iNOS positive cells in cultures as well as nitrite release to the medium in spinal cord neuronal/glial cultures. In addition, this LPS stimulation was effectively attenuated by the presence of an S/B remedy (10 µg/mL). The protein expression levels of inducible nitric oxide synthase (iNOS) or cyclooxygenase (COX)-2 in LPS-stimulated Control or S/B-treated cultures further highlighted the potent anti-inflammatory effects of S/B (Figure 1I,J). Because the S/B remedy contains significant amounts of active flavonoids, the anti-oxidative effect of the S/B remedy was further examined in H_2_O_2_ (1 mM) or tert-BOOH (0.75 mM)-treated spinal cord neuronal/glial cultures. As shown in Figure 2A,B, the free radical levels, determined by fluorescent 2’,7’-Dichlorodihydrofluorescein-reactive oxygen species (DCF-ROS), were markedly increased by a 2 h treatment with H_2_O_2_ or tert-BOOH. The S/B remedy (10 to 200 µg/mL), added to cultures within 10 min after the peroxide treatment, effectively inhibited the peroxide-induced free radical levels at all doses tested (all P < 0.01). Interestingly, the S/B remedy (10 µg/mL) induced sustained increase of protein kiniase C (PKC) phosphorylation and heat shock protein (HSP70) levels when cells were incubated with the S/B remedy for 2 days (Figure 3 and Figure S2). These two molecules are implicated in neuroprotection or cardioprotection [20,21].

### 2.2. Anti-Inflammatory and Anti-Oxidative Effect of S/B Remedy in Microglial Cultures

Oxidative stress was induced by treating microglial cells with two free radical generators (i.e., H_2_O_2_ (3 mM) or tert-BOOH (0.75 mM)), and the antioxidant activity of the S/B remedy was examined by a DCF assay. As shown in Figure 2C, the two free radical generators, H_2_O_2_ and tert-BOOH, significantly induced an increase of DCF-ROS levels in the microglial cells at 2 h after treatment (P < 0.01), and the S/B remedy (80 µg/mL) significantly reduced the peroxide-induced ROS production in the cells (P < 0.01, S/B + H_2_O_2_ vs. H_2_O_2_ alone or S/B + tert-BOOH vs. tert-BOOH alone).

The anti-inflammatory effect of the S/B remedy was subsequently examined in microglia. LPS was added to microglial culture in the presence or absence of the S/B remedy (10 µg/mL) and further incubated for 2 days. Figure 2D,E shows that LPS-induced iNOS protein expression was effectively attenuated by S/B remedy treatment in microglia (*p* < 0.05).

**Figure 4:**
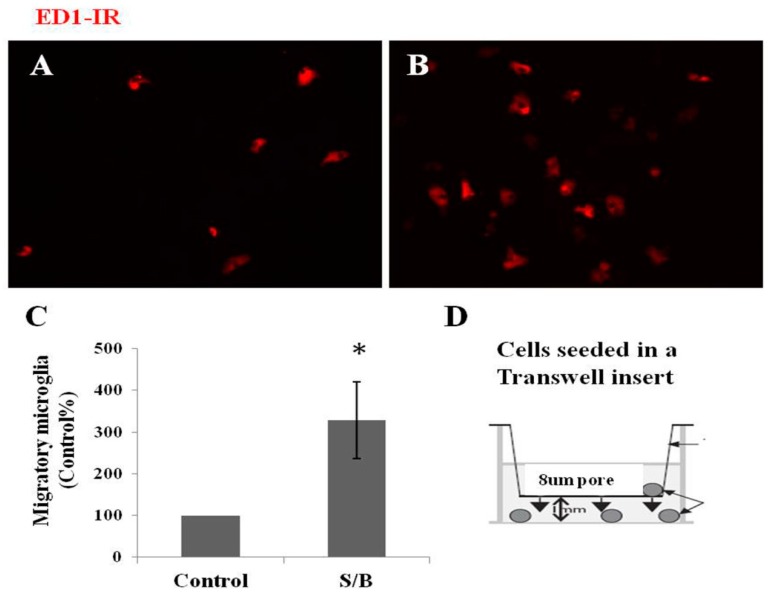
Effect of S/B remedy on microglial migration in mixed glial cultures, wherein (**A**) shows control cultures, ED1-IR microglia, (**B**) shows S/B-treated cultures, ED1-IR microglia, (**C**) shows quantitative results of migratory microglia, and (**D**) refer to a cell-seeded Transwell insert scheme. Mixed glial cells were seeded on 8 µm hanging Transwell inserts inside 24-well plate and maintained in growth medium in the presence or absence of S/B (10 µg/mL) treatment for two days. The migratory microglia in the bottom wells were immunostained and counted. Data are expressed as means ±SEM from three independent experiments. The symbol “*” indicate statistical significance by one-way ANOVA and Bonferroni t-test at *p* < 0.05 (S/B vs. Control group).

The effect of the S/B remedy on the migration of microglia was also examined in mixed glial cultures. Figure 4 shows that numerous ED-1-IR microglias migrated to the lower side of culture plate and the numbers of microglia were significantly enhanced by the presence of the S/B remedy (10 µg/mL).

### 2.3. Systemic Administration of S/B Remedy Inhibited Microglial Activation in the Injured Spinal Cords and Improved Hindlimb Functional Restoration of SCI Rats

**Figure 5:**
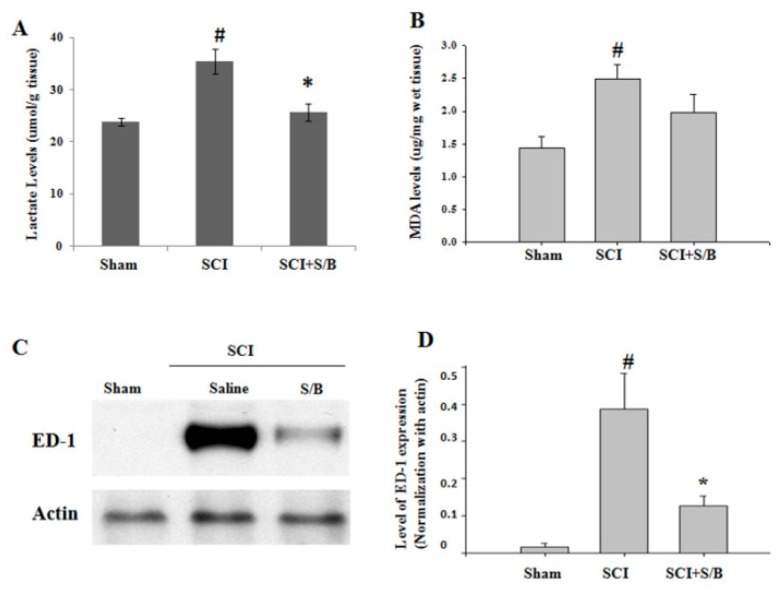
Effects of three consecutive intraperitoneal administration of S/B remedy on injured spinal cords of SCI rats. SCI rats with or without S/B remedy (20 mg/kg) treatment were sacrificed at the third day after SCI, and the injured epicenter of thoracic spinal cords (about 1.5 cm) was rapidly removed and longitudinal dissected into equal 2 segments. One half of the cords was homogenized and processed for measurement of (**A**) lactate or (**B**) lipid peroxidation malondialdehyde (MDA) level. The other half of the cords was homogenized and processed for (**C**) and (**D**) western blot analysis of ED-1 expression. # and * *p* < 0.05 indicate statistical significance as compared with Sham group and SCI group, respectively, by one-way ANOVA and Student–Newman–Keuls Method.

Traumatic spinal cord injury initiates a series of cellular and molecular events. Injury to the spinal cord provokes an inflammatory reaction that results in further tissue damage. Attenuation of the early inflammatory response to spinal cord injury may therefore limit the extent of tissue injury and the consequent disability. Thirty minutes prior to eliciting severe SCI, adult rats were intraperitoneally (ip) injected with 2 or 20 mg/kg of the S/B remedy based on the weight of the rats. After SCI, the rats were daily injected with the S/B remedy at dose of 2 or 20 mg/kg/day for 7 consecutive days. The hindlimb performance of the rats was monitored weekly post-injury (up to 5 weeks) using the open field locomotor test (BBB scale). A group of SCI rats with or without ip administration of S/B remedy (20 mg/kg) were sacrificed at the third day post-injury for analysis of early changes in the injured spinal cords. Because low dose of S/B remedy (2 mg/kg) was without significant effect on rat hindlimb performance in our pilot study, we evaluated the effect of S/B remedy (20 mg/kg, the effective dose) on injured spinal cord at 3 days post-injury. As shown in Figure 5, injury to the spinal cord provoked significant increases of lactate and malondialdehyde (MDA) levels, indicating impaired energy metabolism and free radical damage, respectively. Intraperitoneal injection of S/B remedy (20 mg/kg) to SCI rats for 3 days markedly reduced the lactate level (*p* < 0.05) and had a tendency of decreasing MDA level. Concurrently, SCI induced the expression of ED-1 protein, a marker for activated and phagocytic microglia, in the injured spinal cord, whereas 3 consecutive S/B treatments effectively reduced the ED-1 levels (Figure 5C,D).

**Figure 6:**
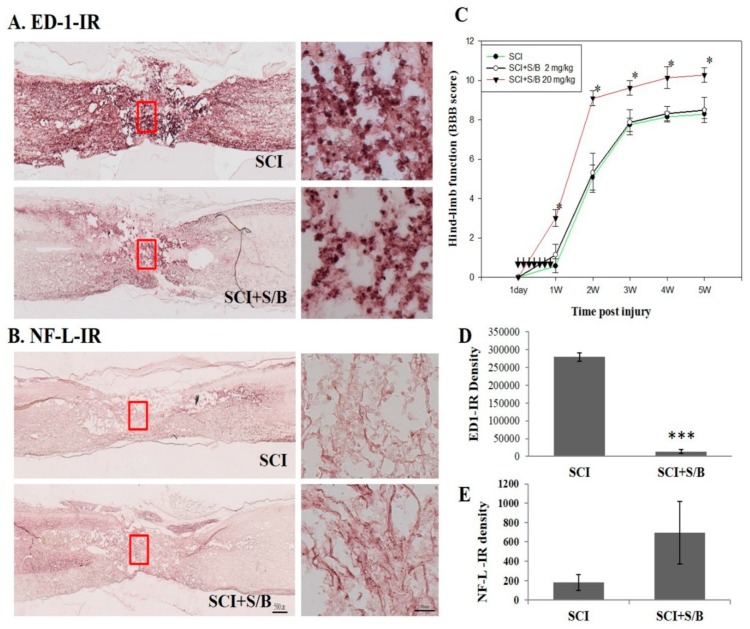
The microscope images of the neurofilament L (NF-L) or ED1-immunoreactive (IR) spinal sections and the hindlimb functional restoration of SCI rats at 5 weeks after S/B remedy treatment, wherein (**A**) refers to the microscope images of ED-1-IR section of SCI or S/B remedy (20 mg/kg)-treated SCI rats, (**B**) refers to the NF-L-IR in the injured spinal cords of two groups of SCI rats. Right images refer to higher magnification of the area marked by the red rectangles as shown in (**A**) and (**B**), respectively, (**C**) refers to the hindlimb functions of SCI rats in each group. Data are expressed as means ± SEM from N = 7, 4, 5 rats for SCI, SCI + S/B 2mg/kg, SCI+S/B 20 mg/kg, respectively, (**D**) and (**E**) refer to the quantitative results of ED-1-IR and NF-L-IR of SCI and S/B (20 mg/kg)-treated rats. Adult SCI rats were intraperitoneally injected with 2 or 20 mg/kg of S/B for 7 consecutive days. The symbol “*” indicate statistical significance by one-way ANOVA and Bonferroni *t*-test for hindlimb function at P < 0.05 (SCI+S/B vs. SCI group). The symbols “***” indicate statistical significance by student t test for NF-L and ED-1 IR at *p* < 0.0001 (SCI+S/B 20 mg/kg vs. SCI).

At 5 weeks post-injury, rats were sacrificed and perfused intravascularly with 4% paraformaldehyde. The thoracic regions of the spinal cords were then sagittally sectioned (10 µm-thick) and processed for immunohistochemical (IHC) staining with anti-neurofilamentt L (NF-L) and ED-1 antibodies (for neurons and activated microglia, respectively). Figure 6A,B shows the result of the IHC staining. Figure 6A shows the microscope images of ED-1-immunoreactive spinal sections of SCI or S/B remedy (20 mg/kg)-treated SCI rats. The right images refer to higher magnification (200×) of the area marked by the red rectangles shown in A. Highly intensed ED-1-positive cells, activated microglia/macrophages, dominated the injured part of the spinal cord, particular the injured epicenter. Treatment with S/B remedy significantly attenuated ED1-IR density in the injured cord (Figure 6A,D). Figure 6B shows the microscope image of NF-L-IR section in the injured spinal cords of two groups of SCI rats. Right images refer to higher magnification of the area marked by the red rectangles shown in B, respectively. The results showed that the spinal axons (neurofilament-positive) had a tendency of being better preserved in the S/B remedy (20 mg/kg)-treated SCI group than in the Sham SCI group, although they did not reach a significant difference (Figure 6B,E). Figure 6C shows the evaluation result of the hindlimb performance for the SCI rats after two doses of five consecutive S/B treatments. The hindlimb performance of the rats was monitored weekly post-injury (up to 5 weeks) using the open field locomotor test (BBB scale, ranging from 0 (no hindlimb movement) to 21 (normal movement-coordinated gait)) [22]. As shown in Figure 6C, intraperitoneal administration of the S/B remedy (20 mg/kg) significantly facilitated hindlimb performance of the SCI rats, whereas S/B remedy treatment at 2 mg/kg was without effect. This also indicates that some active ingredients of the S/B remedy might cross the blood–spinal cord-barrier to exert beneficial functions in SCI rats. A group of SCI rats were treated with the S/B remedy (20 mg/kg) postinjury for 7 consecutive days, the hindlimb behaviors were improved to similar extent as 20mg/kg-treated groups in Figure 6C and Figure S3).

## 3. Discussion

Traumatic SCI initiates primary and secondary injury cascades that cause further permanent damage and neurological dysfunction. Herbal remedies might be promising prescription for spinal cord injury. The present study tested whether the S/B remedy is beneficial in the injured spinal cord. We present evidence supporting the notion that the S/B remedy effectively reduced the extent of inflammation and spinal cord neuronal injury both in vivo and in vitro.

We first demonstrated the beneficial effects of the S/B remedy in primary spinal cord neuron/glial cultures and microglial cultures. Strong anti-oxidative and anti-inflammatory functions of the S/B remedy were observed in these primary cultures. These are consistent with previous in vitro study done by Lin et al. [14]. The in vivo study further demonstrated that the S/B remedy possessed therapeutic potential for the treatment of traumatic SCI. Repeated intraperitoneal injection of the S/B remedy to SCI rats during the acute stage of injury not only attenuated the secondary injury cascade, but significantly enhanced hindlimb behavior restoration in SCI rats. Supporting the observed hindlimb behavioral improvements, the nerve fibers (axons) were preserved with marked reduction of microglial activation in the S/B-treated spinal cords.

Spinal cord tissue is more susceptible to inflammation challenge compared to other CNS regions [23,24]. After SCI, the microglia/macrophages within the epicenter of an injured spinal cord orchestrate inflammatory responses, leading to further devastating neuronal cell death and the loss of oligodendrocytes and myelin. The S/B remedy decreased the number of round/ameboid microglia and the expression of the activated microglial marker ED-1 (CD68) in the injured spinal cords, indicating decreased activation of these cells. Given the fact that the S/B remedy effectively reduced LPS stimulation and peroxide-induced ROS in microglial cultures, the effect of the S/B remedy was more likely to reduce microglial activation in the injured spinal cord. This altered inflammatory response by the S/B remedy at the acute stage of SCI was accompanied by reduced degrees of energy impairment and lipid peroxidation, as evidenced by decreased levels of lactate and malondialdehyde, respectively (Figure 5). Interestingly, the acute S/B remedy treatment to SCI rats was highly effective in prolonged reduction of microglial activation in the injured spinal cord even at five weeks after injury (Figure 6A).

Compared to other tissues, the CNS is more susceptible to oxidative damage because it has high levels of polyunsaturated lipids and possesses a high rate of oxidative metabolic activity [25,26,27]. Hydrogen peroxide can be over-produced during the pathological process of acute neuronal toxicity [28]. Endogenous H_2_O_2_ mainly originates from the enzymatic or spontaneous dismutation of superoxide anions, which are byproducts of cellular oxidases [29,30]. Due to its high membrane permeability, exogenous H_2_O_2_ may enter cells immediately after exposure [26,31]. In the present study, we employed H_2_O_2_ or tert-BOOH to cultured neuronal/glial cells or microglia to induce oxidative stress that could be markedly attenuated by the S/B remedy, indicating a potent antioxidant property of S/B. Consistently, the S/B remedy also induced PKC phosphorylation and increased HSP70 level (Figure 3). HSP70 is a 70 kDa stress protein and mediates neuroprotection induced by ischemic preconditioning [20]. Furthermore, HSP70 induction has been reported to ameliorate pathological changes in neurodegenerative diseases and inflammation [21,32]. On the other hands, activation of PKC was reported to participate in ischemia preconditioning, and induced cardioprotection in several animal models [33,34]. Baicalein and baicalin was reported to alleviate liver injury with the involvement of PKC phosphorylation [35]. The S/B remedy was analyzed by high performance liquid chromatography (HPLC) with UV detection by Chen et al. [9] and in our pilot study (Figure S1): it contains significant amounts of baicalin and baicalein. Several lines of evidence have demonstrated antioxidative activities of baicalein or baicalin, such as inhibiting peroxide or iron-induced lipid peroxidation [19,36]. The antioxidative effect of S/B remedy on peroxide toxicity in culture or on contusive spinal injury was, at least in part, attributed to the abundance of baicalein and baicalin in the S/B remedy.

The main causes of dysfunction after SCI include primary and secondary injuries [37,38]. Secondary injury after SCI includes inflammation, glial cell activation and scar tissue formation, which will affect the regenerating nerve microenvironment. Following damage to the spinal cord, an inflammatory process is initiated by the activation of resident microglia and astrocytes as well as infiltrating peripheral macrophages and lymphocytes. Our data clearly demonstrated that systemic administration of the S/B remedy to the SCI rats resulted in down-regulation of the microglial activation in the injured epicenter of spinal cord. This implies that ingredient(s) of the S/B remedy might enter the blood–spinal cord barrier to exert beneficial function. However, it is not yet known what components of the S/B remedy contribute to this effect. The composition of the S/B remedy is not as complicated as those of Sho-Saiko-To. Further works needed to be done considering an oral administration of the S/B remedy and/or with methylprednisolone co-treatment in SCI victims.

In conclusion, the present work discover that an herbal composition prepared from the roots of *bupleurum* and *scutellaria* exhibits neuroprotective effects and improves functional recovery in SCI rats. We demonstrated that the S/B remedy can modulate the macrophage/microglial activation to SCI to preserve nerve tissues and improve hindlimb performance. Thus, S/B may become a promising therapeutic agent for SCI patients in the future. More studies are needed to confirm the effectiveness, the therapeutic window, and the dosages after SCI.

## 4. Materials and Methods

### 4.1. Reagents and Antibodies

2′,7′-Dichlorodihydrofluorescein diacetate (DCF-DA) was obtained from Molecular Probe (Eugene, OR, USA). Lipopolysaccharide (LPS; E. coli 0111:B4), hydrogen peroxide (H_2_O_2_) and tert-butyl hydroperoxide (t-BOOH) were purchased from Sigma-Aldrich. Other reagents were purchased from Sigma-Aldrich unless stated otherwise. Cultured media, serum-free supplements and antibiotics were purchased from Gibco (Carlsbad, CA, USA). Tissue culture plastics were from BD Bioscience (San Jose, CA, USA). Primary antibodies and suppliers were: rabbit or mouse anti-βIII tubulin (Covance), mouse anti-ED1 (CD68, for activated and phagocytic microglia/macrophage, Serotec, England), rabbit anti-phospho PKC (CS9371,Cell signaling technology, CO, USA), mouse anti-inducible nitric oxide synthase (iNOS, BD Bioscience, CA, USA), and goat anti-actin and anti-HSP70 (Santa Cruz Biotech, Dallas, TX, USA).

### 4.2. Preparation of S/B Remedy

The S/B remedy was a hot water extract of *Scutellaria baicalensis Georgi* (Sb) and *Bupleurum scorzonerifolfium Willd* (Bs) which were obtained from a local wholesale distributor. The preparation method for S/B and storage followed the method described in Lin et al. [14]. Briefly, 18 g Sb and 42 g Bs were extracted by 1.8 L boiling water until volume reduction to 1 L. The extract was subsequently filtered and lyophilized.

### 4.3. Animals

Sprague-Dawley (SD) rats were obtained from the Animal Center of National Yang-Ming University or National Science Council, Taiwan. Primary neuron-glial cultures or microglial cultures were prepared from embryonic or fetal SDF rats. Female adult SD rats ranging from 240 to 280 g were used for induction of contusive SCI models. Animal handling and experimental protocols were carefully reviewed and approved by the animal studies committee of Taipei Veteran General Hospital (IACUC 2014-004 and IACUC 2015-167).

### 4.4. Neuronal/Glial Cultures

Mixed neuron/glia cells cultures were prepared from spinal regions of embryonic Sprague-Dawley rat fetus at gestation day 15 as described in Tsai et al. [39,40]. Briefly, cells were dissociated with mixtures of papain/protease/deoxyribonuclease I (0.1%: 0.1%: 0.03%) and plated onto poly-lysine-coated dishes at a density 1–2 × 10^5^ cells/cm^2^. Cells were maintained in Dulbecco’s modified Eagle’s medium (DMEM, Thermo Fisher Scientific, Waltham, MA, USA) supplemented with 10% fetal bovine serum (FBS). Mixed neuron/glial cultures were treated with S/B in the presence or absence of toxins at 2nd or 3rd day after cell seeding. Tert-butyl hydroperoxide (BOOH), Hydrogen peroxide (H_2_O_2_), and LPS were used to induce oxidative stress and inflammation in cultures.

### 4.5. Mixed Glial Cultures and Microglial Cultures

Mixed glial cultures were prepared from cerebral cortices of new bone SD rat pups, as described previously [41,42]. Briefly, triturated neonatal cortex was passed through nylon clothes (80 and 10 µm), plated in flasks and maintained in DMEM supplemented with 10% FBS. The cells were incubated at 37 °C in a water-saturated atmosphere of 5% CO_2_/95% air. Experiments were conducted after subculture of the confluent glial cultures and seeded on 8 µm hanging Transwell inserts (Millipore, Watford, UK) inside 24-well plate and maintained in growth medium with or without S/B (10 µg/mL) treatment. Two days later, the migratory microglias in the bottom wells were immunostained and counted. Microglial cultures were purified from confluent mixed glial cultures as previously described [24]. Briefly, floating cells and weakly attached cells in mixed glial cultures were isolated by shaking the flasks for 2 h at 180 rpm. The resulted cell suspension was collected, pelleted and reseeded to culture dish. After seeding for 30 min, the unattached cells were removed. The strongly adhering cells were microglia. Enriched microglia were > 95% immunoreactive for ED-1.

### 4.6. Spinal Cord Contusion and Treatment

Contusive SCI was induced using the NYU weight-drop device which was developed by New York University (NYU). Female adult SD rats were anesthetized and dorsal laminectomy was carried out at the level of the ninth thoracic (T) vertebra. The dorsal surface of T9-T10 spinal cord was injured by dropping a 10 g rod from a height of 50 mm. Adult rats were intraperitoneally (ip) injected with 2 or 20 mg/kg of Sb/Bs 30 min prior to eliciting severe SCI. Afterwards, S/B was administered daily at a dosage of 2 or 20 mg/kg/day for 7 consecutive days. To avoid urinary tract infections, manual emptying of the urinary bladder was carried out twice daily. The hindlimb functions of SCI rats were monitored at day 2 and weekly post-injury with the locomotor’s rating Basso, Beattie, Bresnahan (BBB) open field scale, ranging from 0 (no hindlimb movement) to 21 (normal movement-coordinated gait). At 5 weeks postinjury, animals were sacrificed for histological studies. SCI rats with or without S/B (20 mg/kg, 3 consecutive i.p. injections) treatment were also sacrificed at the third day post-injury for biochemical and western blot analysis of the spinal cords. The injured epicenter of thoracic spinal cords (about 1.5 cm) was rapidly removed and longitudinal dissected into equal 2 segments. The one half of each cord was homogenized in ice-cold PBS buffer containing 5 mM BHT by sonication and processed for measurement of lactate or lipid peroxidation malondialdehyde (MDA) level. The other half of each cord was homogenized in lysis buffer and was processed for western blot analysis for ED-1 (for activated and phagocytic microglia/macrophage).

### 4.7. Biochemical Assays

For detection of free radical ROS, cultures were pre-loaded with 2′,7′-DCF-DA for 30 min and treated with H_2_O_2_ or BOOH in the presence or absence of Sb/Bs for 2 h. The preloaded DCFH was rapidly oxidized in the presence of oxygen free radicals to highly fluorescent 2′,7′-DCF (oxidised form) and the resulted fluorescent DCF-ROS levels were measured by fluorescence plate reader. The production of nitric oxide (NO) was assayed as an accumulation of nitrite in medium using colorimetric Griess assay as described previously [24]. Cell death was evaluated by the release of lactate dehydrogenase (LDH) after neuronal injury. LDH activity in the medium was measured using a kit from Promega (Madison, WI, USA). Lactate levels in the spinal cord were assayed using a commercial kit from sigma. Measurement of malondialdehyde (MDA), an indicator of lipid peroxidation, was conducted in spinal cords using a commercial kit (BIOXYTECH LPO-586, OXIS Health Products, Inc., Portland, OR, USA).

### 4.8. Western Blot Analysis

After experimental periods, cultures were washed twice with PBS and solubilized in a lysis buffer containing 40 mMTris buffer (pH 7.5), 7M urea/2M thiourea, 4% CHAPS, 1 mM PMSF, 1 mM Na3VO4,1 mM dithiothreitol, and a protease inhibitor kit (BM, Mannheim, Germany). Rat spinal cord segments (T8–10) were homogenized in the same lysis buffer (500 µL) with the help of a sonicator. Equal amounts of proteins of the homogenates were analyzed by Western blot, using sodium dodecyl sulfate-polyacrylamide gel electrophoresis (SDS-PAGE) 8% or 12% gels, as previously described [24,40].

### 4.9. Histological Examination of Spinal Cord Injury

Rats were over anesthetized and transcardially perfused with normal saline, followed by a 4% paraformaldehyde solution. After postfixation and cryosection of the tissue, spinal cord sections were processed for immunostaining with primary antibodies against NF-L (for all axons) and ED1 (for activated microglia). The tissue sections were further incubated with respective secondary antibodies for histological evaluation as previously described [43,44].

## Supplementary Materials

The following are available online, Figure S1: Analysis of S/B remedy by HPLC with UV detection; Figure S2: Effects of S/B remedy on levels of phosphoPKC (pPKC) and HSP70 in microglia cultures; Figure S3: Effects of S/B remedy on the hindlimb functions of spinal cord injured rats.
