# Supplementary material for: Characterizing the Neuroprotective Effects of S/B Remedy (Scutellaria baicalensis Georgi and Bupleurum scorzonerifolfium Willd) in Spinal Cord Injury
Source: Molecules. 2019 May 16;24(10):1885. doi: 10.3390/molecules24101885 (PMC6571778; doi:10.3390/molecules24101885)
Supplement: Supplementary file 1 [file molecules-24-01885-s001.pdf]

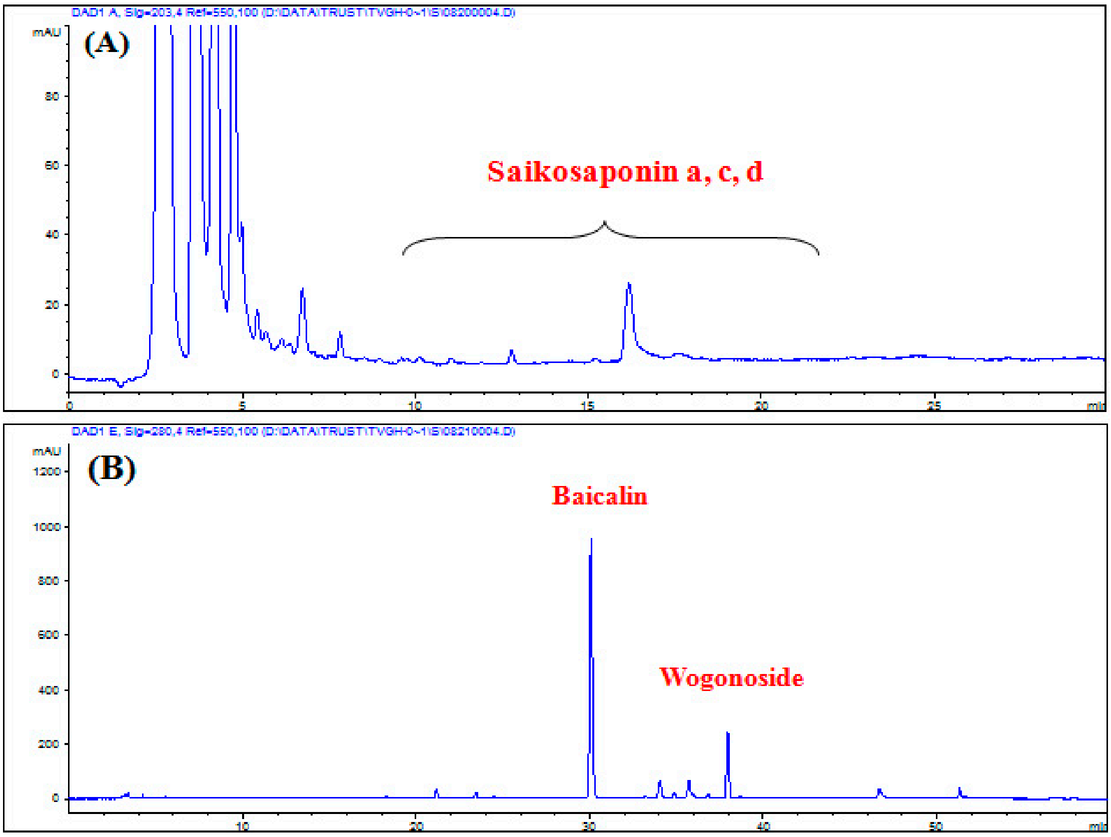

Supplementary Figure1 Analysis of S/B remedy by HPLC with UV detection.Two gradient programs were used for eluting the compounds in S/B with mobile phase containing (a)20 mM KH_2PO_4 and (b) acetonitrile. Column: COSMOSIL 5C18-AR-II column (4.6 I.D.×250 mm). Flow rate: 1.0 mL/min.(A) HPLC chromatograph run by gradient program I with detection at UV wavelength of 203 nm.(B) HPLC chromatograph run by gradient program II with detection at UV 280 nm.

Program I:

Time (min)	a%	b%	Curve
0	70	30	*
25	30	70	Linear
30	70	30	Linear

Program II:

Time (min)	a%	b %	Curve
0	90	10	*
30	75	25	Linear
40	65	35	Linear
55	25	75	Linear
60	90	10	Linear

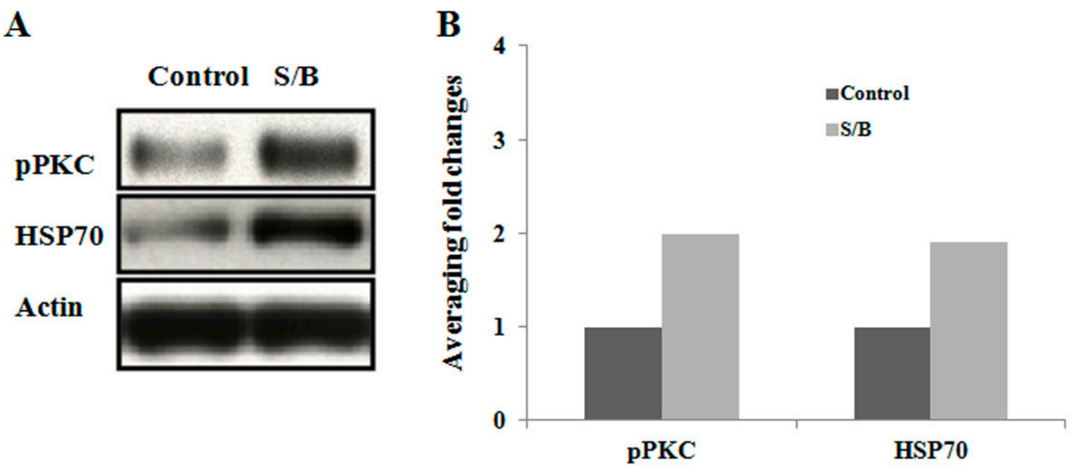

Supplementary Figure2 Effects of S/B remedy on levels of phosphoPKC (pPKC) and HSP70 in microglia cultures**,** wherein (A) shows the protein expression of control or S/B-treated cultures (B) the quantitative results of pPKC or HSP70 expression levels in A. Data are expressed as means from 2 independent experiments.

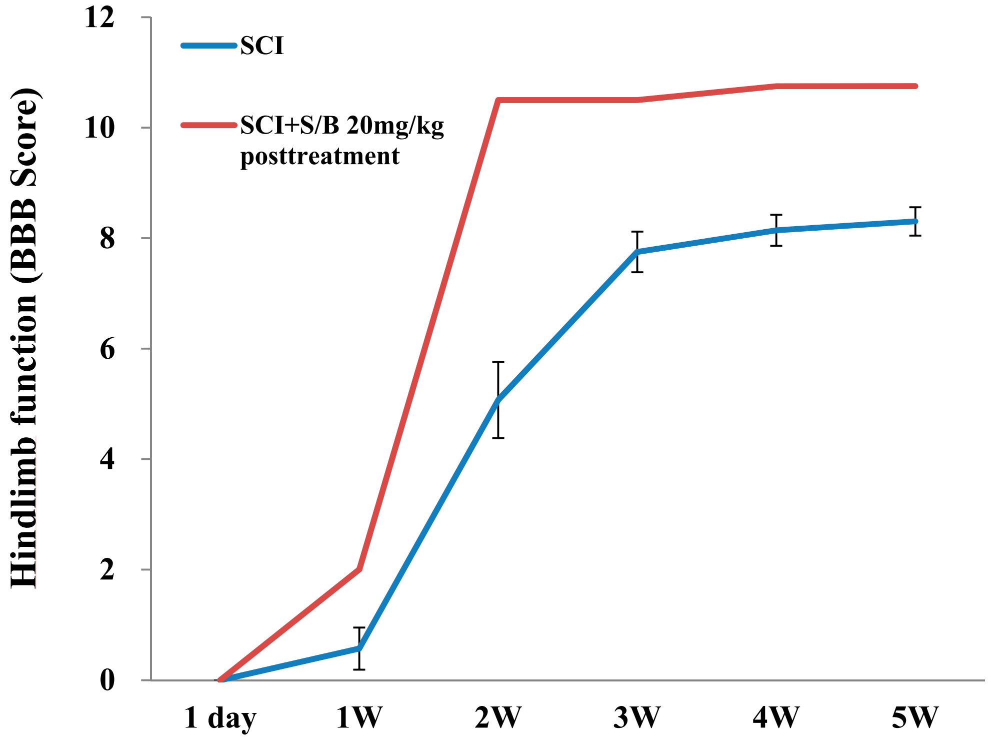

Supplementary Figure3. Effects of S/B remedy on the hindlimb functions of spinal cord injured rats**.** S/B remedy was intraperitoneally injected to SCI rats within one hour after injury and daily for 7 consecutive days.
